# The Polo kinase Cdc5 is regulated at multiple levels in the adaptation response to telomere dysfunction

**DOI:** 10.1093/genetics/iyac171

**Published:** 2022-11-07

**Authors:** Héloïse Coutelier, Oana Ilioaia, Jeanne Le Peillet, Marion Hamon, Damien D’Amours, Maria Teresa Teixeira, Zhou Xu

**Affiliations:** Sorbonne Université, PSL, CNRS, UMR8226, Institut de Biologie Physico-Chimique, Laboratoire de Biologie Moléculaire et Cellulaire des Eucaryotes, 75005 Paris, France; Sorbonne Université, CNRS, UMR7238, Institut de Biologie Paris‐Seine, Laboratory of Computational and Quantitative Biology, 75005 Paris, France; Sorbonne Université, CNRS, UMR7238, Institut de Biologie Paris‐Seine, Laboratory of Computational and Quantitative Biology, 75005 Paris, France; Sorbonne Université, CNRS, UMR7238, Institut de Biologie Paris‐Seine, Laboratory of Computational and Quantitative Biology, 75005 Paris, France; Sorbonne Université, PSL, CNRS, FR550, Institut de Biologie Physico-Chimique, 75005 Paris, France; Ottawa Institute of Systems Biology, Department of Cellular and Molecular Medicine, University of Ottawa, Ottawa, ON K1H 8M5, Canada; Sorbonne Université, PSL, CNRS, UMR8226, Institut de Biologie Physico-Chimique, Laboratoire de Biologie Moléculaire et Cellulaire des Eucaryotes, 75005 Paris, France; Sorbonne Université, CNRS, UMR7238, Institut de Biologie Paris‐Seine, Laboratory of Computational and Quantitative Biology, 75005 Paris, France

**Keywords:** adaptation to DNA damage, Polo kinase Cdc5, PP2A, DNA damage checkpoint, telomere

## Abstract

Telomere dysfunction activates the DNA damage checkpoint to induce a cell cycle arrest. After an extended period of time, however, cells can bypass the arrest and undergo cell division despite the persistence of the initial damage, a process called adaptation to DNA damage. The Polo kinase Cdc5 in *Saccharomyces cerevisiae* is essential for adaptation and for many other cell cycle processes. How the regulation of Cdc5 in response to telomere dysfunction relates to adaptation is not clear. Here, we report that Cdc5 protein level decreases after telomere dysfunction in a Mec1-, Rad53- and Ndd1-dependent manner. This regulation of Cdc5 is important to maintain long-term cell cycle arrest but not for the initial checkpoint arrest. We find that both Cdc5 and the adaptation-deficient mutant protein Cdc5-ad are heavily phosphorylated and several phosphorylation sites modulate adaptation efficiency. The PP2A phosphatases are involved in Cdc5-ad phosphorylation status and contribute to adaptation mechanisms. We finally propose that Cdc5 orchestrates multiple cell cycle pathways to promote adaptation.

## Introduction

In response to DNA damage, cells ensure genome stability by repairing the initial injury. The DNA damage checkpoint (DDC) detects and processes the damage, and arrests the cell cycle to provide time for repair. The coordination between the cell cycle and the repair pathways ensured by the DDC is essential to prevent chromosome segregation with unrepaired damage. When the cell fails to repair the damage, a process called adaptation to DNA damage allows the bypass of the checkpoint arrest and completion of mitosis despite the presence of unrepaired DNA damage, thus inducing genome instability (Sandell and Zakian 1993; Toczyski *et al.* 1997; Lee *et al.* 1998; Galgoczy and Toczyski 2001). In unicellular eukaryotes, adaptation may be viewed as a survival strategy for cells experiencing unrepairable damage that can be asymmetrically segregated into daughter cells or be dealt with in the next cell cycle (Galgoczy and Toczyski 2001; Kaye *et al.* 2004; Coutelier *et al.* 2018; Roux *et al.* 2021). In mammals though, adaptation may contribute to tumor emergence and progression by evading checkpoint control and promoting genome instability (Strebhardt and Ullrich 2006; Gutteridge *et al.* 2016). While the mechanisms and factors involved in the DDC and repair pathways have been studied for decades, our understanding of adaptation to DNA damage is more limited.

In the model organism *Saccharomyces cerevisiae*, a double-strand break (DSB) is signaled by the phosphatidylinositol-3′-kinase-like kinases (PIKK) Tel1 and Mec1. Mec1 phosphorylates the adaptor protein Rad9 and the kinases Rad53 and Chk1, which are recruited to Rad9. The full activation of Rad53 requires an additional autohyperphosphorylation step. Both Rad53 and Chk1 activation promote the stability of securin Pds1 to prevent chromosome segregation (Cohen-Fix and Koshland 1997; Sanchez *et al.* 1999; Agarwal *et al.* 2003). Phosphorylated Rad53 inhibits the mitotic exit network (MEN) by targeting the Polo kinase Cdc5 (Cheng *et al.* 1998; Sanchez *et al.* 1999) and triggers a transcriptional response, which includes the direct inhibition of the transcriptional activator Ndd1, controlling the expression of the *CLB2* cluster of mitotic genes (Spellman *et al.* 1998; Gasch *et al.* 2001; Jaehnig *et al.* 2013; Edenberg *et al.* 2014; Yelamanchi *et al.* 2014). When no repair is possible, cells stay arrested for 4–16 h but then undergo adaptation to DNA damage and alleviate the DDC arrest by returning Rad53 and Chk1 to their unphosphorylated state (Lee *et al.* 2000; Pellicioli *et al.* 2001), while maintaining the upstream part of the DDC largely intact (Melo *et al.* 2001; Donnianni *et al.* 2010; Vidanes *et al.* 2010). Besides DSBs, telomere dysfunction is perceived by the cell as a persistent DNA damage and has been widely used as a model to study the DDC and adaptation. Telomere dysfunction can be induced by the conditional loss of Cdc13, an essential factor for telomere protection, which is achieved by placing the *cdc13-1* mutant at restrictive temperature to generate resected telomeres and activate the DDC (Garvik *et al.* 1995; Toczyski *et al.* 1997).

Cdc5 is an essential kinase of the cell cycle regulating a variety of processes in mitosis and cytokinesis (Botchkarev and Haber 2018). Cdc5’s expression starts at S phase and increases in the later phases of the cell cycle until cytokinesis (Charles *et al.* 1998; Cheng *et al.* 1998; Shirayama *et al.* 1998), consistent with *CDC5* belonging to the cell cycle-regulated *CLB2* cluster of genes, whose coordinated expression strongly depends on the transcription factor complex consisting of Mcm1, Fkh2, and Ndd1 (coactivator) or Isw2 (corepressor) (Spellman *et al.* 1998; Koranda *et al.* 2000; Zhu *et al.* 2000; Sherriff *et al.* 2007). Phosphorylation by the cyclin-dependent kinase 1 (Cdk1) is required to stabilize Cdc5 (Crasta *et al.* 2008; Simpson-Lavy and Brandeis 2011). After mitosis, Cdc5 is ubiquitinated by the anaphase-promoting complex (APC) associated with Cdh1 and targeted for proteasomal degradation (Charles *et al.* 1998). The kinase activity of Cdc5 depends on phosphorylation by Cdk1 and potentially other kinases at several sites, including amino acids T70, T238, and T242 (Mortensen *et al.* 2005; Rawal *et al.* 2016; Rodriguez-Rodriguez *et al.* 2016). Among the several mutants of adaptation that have been identified over the past 2 and a half decades (Harrison and Haber 2006; Serrano and D'Amours 2014), a point mutant allele of *CDC5*, called *cdc5-ad* (for “adaptation-defective”; L251W), is specific for adaptation and does not seem to affect most other functions of Cdc5 in a normal cell cycle (Toczyski *et al.* 1997; Charles *et al.* 1998; Rawal *et al.* 2016). Besides, overexpression of Cdc5 accelerates adaptation, suggesting that Cdc5 plays a promoting role in adaptation (Sanchez *et al.* 1999; Hu *et al.* 2001; Donnianni *et al.* 2010; Vidanes *et al.* 2010). However, despite its central role in adaptation, how Cdc5 is regulated in response to DNA damage is not well characterized. In studies on global gene expression in response to alkylating agent methyl methane sulfonate (MMS) treatment and gamma irradiation, *CDC5* transcripts were found to be decreased in a *MEC1*- and *RAD53*-dependent manner (Gasch *et al.* 2001; Jaehnig *et al.* 2013; Edenberg *et al.* 2014). In contrast, Cdc5’s protein level and kinase activity in response to MMS or telomere dysfunction have not been conclusively established as reports differed in their experimental conditions and conclusions (Charles *et al.* 1998; Cheng *et al.* 1998; Hu *et al.* 2001; Maas *et al.* 2006; Zhang *et al.* 2009; Vidanes *et al.* 2010; Ratsima *et al.* 2016; Rawal *et al.* 2016).

Here, we investigate the regulation of Cdc5 and the adaptation-defective mutant Cdc5-ad in response to telomere dysfunction. We find that the DDC induces an Mec1/Rad53- and Ndd1-dependent downregulation of Cdc5 expression, which is critical to prevent cells from adapting prematurely. We also identify phosphorylation sites of Cdc5/Cdc5-ad that are involved in the adaptation response to telomere dysfunction. We finally propose that Cdc5 acts in multiple pathways to control adaptation, which might explain the complexity of Cdc5’s regulation in response to telomere dysfunction.

## Materials and methods

### Yeast strains and plasmids

All strains are from the W303 background (*ura3-1 trp1-1 leu2-3,112 his3-11,15 can1-100*) corrected for *RAD5* and *ADE2* (Supplementary Table 1). Most contain the *cdc13-1* allele and were grown at the permissive temperature of 23°C in yeast extract, peptone, dextrose media and at 32°C to induce telomere dysfunction. Overexpression and deletion strains were created using PCR-based methods as described in (Longtine *et al.* 1998). Point mutations were performed using Cas9-mediated gene targeting as described in (Anand *et al.* 2017). To ectopically express *CDC5* or *cdc5-ad* from a 2 µ plasmid, the genomic coding sequence and 500 bp of the 5′ and 3′ UTR were amplified by colony PCR and cloned into the *SacI*-*BamHI* sites of 2 µ plasmid pRS42H.

### Microcolony assay

Microcolony assays were performed using the *cdc13-1* mutant to assess adaptation, as described in (Toczyski 2006). Briefly, telomere dysfunction was induced in exponentially growing cells by incubation at 32°C for 3 h. A hundred microliters of a culture at OD_600 nm_ = 0.1 were then plated on a prewarmed plate. Plates were visualized on a dissection microscope (MSM System 400, Singer Instruments) immediately at 3 and 24 h. At each position, the number of cell bodies was counted and microcolonies were defined as comprising ≥3 cell bodies. Two-sided *t*-tests were used to assess statistical significance with a 0.05 threshold for the *P*-value.

### SDS-PAGE and western blot analysis

Aliquots of 5 × 10^7^ cells were harvested by centrifugation. The pellets were lysed in 0.2 M NaOH on ice for 10 min and proteins were precipitated by the addition of 50 µl of 50% trichloroacetic acid. The samples were centrifuged at 16,100 × *g* for 10 min at 4°C and the pellets were resuspended in 4× Laemmli buffer and heated for 5 min at 95°C. Samples were separated in a denaturing 7.5% 37.5:1 polyacrylamide gel, and proteins were transferred to a nitrocellulose membrane (Amersham Protran 0.45 NC, GE Healthcare). The membranes were stained with Ponceau Red and immunoblotted with anti-Rad53 antibody (EL7.E1; Abcam), which recognizes both the unphosphorylated and phosphorylated forms of Rad53, anti-Cdc5 antibodies (11H12 and 4F10; Medimabs), anti-HA antibody (3F10; Roche), and anti-Pgk1 antibody (22C5D8; Abcam). Blots were then incubated with a horseradish peroxidase-coupled secondary antibody, and the signal was detected using ECL reagent (Amersham, GE Healthcare). All western blots were independently replicated in the laboratory.

### Immunoprecipitation

Immunoprecipitation was performed as in Cohen *et al.* (2011) with minor modifications. After growth in the different conditions described in the results, ∼10^9^ cells were harvested, washed in Milli-Q water and resuspended in lysis buffer [50 mM Tris-HCl, pH 8.0, 150 mM NaCl, 0.6% Triton X-100, 10% glycerol, protease inhibitors (cOmplete Mini EDTA-free Protease Inhibitor Cocktail, Roche), and phosphatase inhibitors (Phosphatase Inhibitor Cocktail Set II, Millipore)]. Cells were lysed with glass beads in a cooled FastPrep (MP Biomedicals). Cell lysates were cleared by centrifugation at 13,000 × *g* for 30 min and supernatants were incubated for 30 min with 2.5 µg/sample anti-HA antibodies (clone 3F10; Roche) bound to 50 µl/sample (or 1.5 mg/sample) protein G-coated magnetic beads (Dynabeads Protein G; Invitrogen). The subsequent steps of immunoprecipitation and washes were performed according to the manufacturer’s instructions. The immunoprecipates were eluted in 2× Laemmli buffer.

### Mass spectrometry analysis

After separation by SDS-PAGE, proteins were fixed and revealed by silver nitrate staining according to the protocol described in Rabilloud (2012). Bands of interest were excised and prepared for trypsin digestion. Briefly, proteins were destained with a freshly prepared solution containing 15 mM potassium ferricyanide and 50 mM sodium thiosulfate. Proteins were reduced by 10 mM dithiotreitol in 50 mM ammonium bicarbonate (AMBIC) for 30 min at 56°C and further alkylated by incubation in the dark with 50 mM iodoacetamide in 50 mM AMBIC. Then, protein samples were digested overnight at 37°C with 125 ng modified porcine trypsin (Trypsin Gold, Promega). Peptides were extracted under acidic conditions and dried out using a speedvac concentrator. Then, peptide mixtures were resuspended in 10 µl of solvent A [0.1% (v/v) formic acid in 3% (v/v) acetonitrile] and frozen at −20°C until use.

Mass spectrometry analyses were performed on a Q-Exactive Plus hybrid quadripole-orbitrap mass spectrometer (Thermo Fisher, San José, CA, USA) coupled to an Easy 1000 reverse phase nano-flow LC system (Proxeon) using the Easy nano-electrospray ion source (Thermo Fisher). Five microliters of peptide mixture were loaded onto an Acclaim PepMap precolumn (75 µm × 2 cm, 3 µm, 100 Å; Thermo Scientific) equilibrated in solvent A and separated at a constant flow rate of 250 nl/min on a PepMap RSLC C18 Easy-Spray column (75 µm × 50 cm, 2 µm, 100 Å; Thermo Scientific) with a 90-min gradient {0–20% B solvent [0.1% (v/v) formic acid in acetonitrile] in 70 min and 20–37% B solvent in 20 min}.

Data acquisition was performed in positive and data-dependent modes. Full scan MS spectra (mass range *m*/*z* 400–1,800) were acquired in profile mode with a resolution of 70,000 (at *m*/*z* 200) and MS/MS spectra were acquired in centroid mode at a resolution of 17,500 (at *m*/*z* 200). All other parameters were kept as described in Perez-Perez *et al.* (2017).

Protein identification was performed with the MASCOT software (version 4; Matrix Science, London, UK) via the Proteome discoverer software (version 2.2; Thermo Scientific) with *S. cerevisiae* UniprotKB protein database. The search parameters were set as followed: 2 missed cleavages allowed cysteine carbamidomethylation as fixed modifications and methionine oxidation, N Ter protein acetylation, and phosphorylation on serine, tyrosine, and threonine as variable modifications with a peptide mass tolerance of 10 ppm. False discovery rate for protein identification was fixed at 0.01.

### RNA extraction and quantitative reverse-transcriptase PCR

5 × 10^7^ cells were harvested, washed in Milli-Q water, and frozen. Frozen cell pellets were incubated in the presence of lyticase for 30 min at 30°C to generate spheroplasts. Total RNA was extracted using the RNeasy mini kit (Qiagen) and following the manufacturer’s instructions. To eliminate genomic DNA contamination, an additional DNase treatment was performed using Turbo DNA-free kit (Invitrogen). The extracted RNA was quantified and quality controlled using a NanoDrop One spectrophotometer (ThermoFischer Scientific). Six hundred nanograms of total RNA was reverse-transcribed into cDNA in a 20-μl reaction using the SuperScript II Reverse Transcriptase kit (Invitrogen). Real-time PCR was performed using Fast SYBR Green Master Mix (Applied Biosystems) on CFX96 Real-Time System instrument (Bio-Rad). The PCR program consisted of 1 hold at 95°C for 10 min, followed by 40 cycles of 15 s at 95°C and 30 s at 57°C. Quantification of the quantitative PCR (qPCR) data was performed using the ΔΔCt method (Livak and Schmittgen 2001) using *ACT1* transcript as a reference: primers for *CDC5*: 5′-TGGCAATATCCGACGGAGG-3′ (forward) and 5′-GGATATCCTGGGATCTCGC-3′ (reverse) and primers for *ACT1*: 5′-CTGGTATGTGTAAAGCCGGT-3′ (forward) and 5′-ACGTAGGAGTCTTTTTGACCCA-3′ (reverse), corresponding to p32 and p33, respectively, from Mozdy and Cech (2006).

### Time-lapse microscopy

Exponentially growing cells at OD_600 nm_ = 0.2 were injected into the microfluidics chambers of a CellASIC plate (Y04C-02 plate for haploid cells, CellASIC ONIX2 Microfluidic System; Millipore), through the inlet wells. A constant flow of rich media (20 µl/h for 0.036-µl chambers) fed the chambers, which were kept at the constant temperature of 32°C. Cells in the microfluidic device were imaged using a fully motorized Axio Observer Z1 inverted microscope (Zeiss) with a 100× immersion objective, a Hamamatsu Orca R2 camera, and constant focus maintained with focus stabilization hardware (Definite focus, Zeiss). The temperature was maintained at 32°C with a controlled heating unit and an incubation chamber. Images were acquired every 10 min using ZEN software (Zeiss). All aspects of image acquisition were fully automated and controlled, including temperature, focus, stage position, and time-lapse imaging.

## Results

### Cdc5 protein level decreases in response to telomere dysfunction

**Fig. 1.**
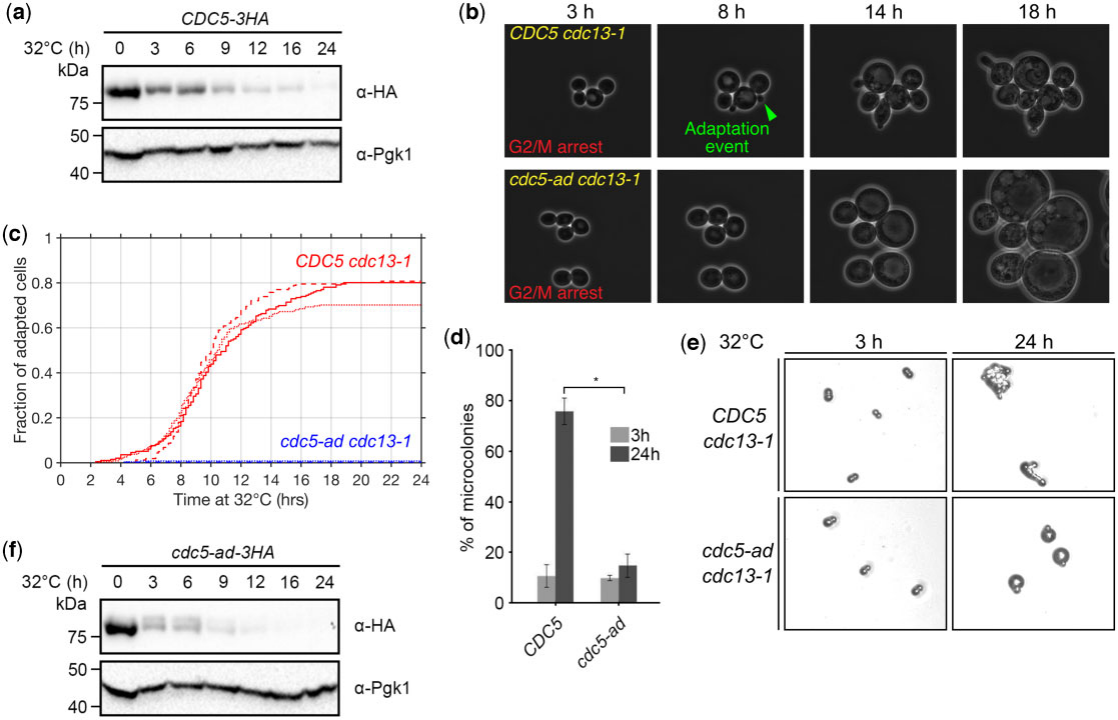
Adaptation response to telomere dysfunction at the cellular and protein level. a) Representative western blot of Cdc5 in a time course experiment after induction of telomere dysfunction by incubating *cdc13-1* cells at 32°C. Pgk1 is shown as a loading control. b) Sequential microscopy images of *CDC5* and *cdc5-ad* cells monitored in microfluidics chambers, used for the analysis shown in (c). c) Cumulative curves of the fraction of adapted cells as a function of time after induction of telomere dysfunction, obtained from microfluidic analysis of adaptation events at the single cell level, in *CDC5 cdc13-1* and *cdc5-ad cdc13-1* cells. Three independent experiments for each strain are shown in continuous line, dashed line, and dots. d) Microcolony assay measuring the fraction of microcolonies formed by *CDC5 cdc13-1* and *cdc5-ad cdc13-1* cells at 32°C at 3 and 24 h. Data are presented as means ± SD of *N* = 3 independent experiments. *n* ≥ 150 cells for each condition. e) Representative pictures of isolated G2/M cells at 3 h and unadapted cells (*cdc5-ad*) or microcolonies of ≥3 cell bodies (*CDC5*) at 24 h, after telomere dysfunction. Used to perform microcolony assays. f) Representative western blot of Cdc5-ad in a time course experiment after induction of telomere dysfunction by incubating *cdc13-1* cells at 32°C. Pgk1 is shown as a loading control.

To understand how Cdc5 is regulated in response to DNA damage, we induced telomere deprotection by incubating *cdc13-1* cells at the restrictive temperature of 32°C and analyzed C-terminally 3xHA-tagged Cdc5 proteins by western blot at different time points (Fig. 1a). We observed that the amount of Cdc5 decreased from 3 h onward compared to time point 0. This result was confirmed in a strain with an untagged Cdc5, using an anti-Cdc5 antibody for western blot (Supplementary Fig. 1a). As a control, we confirmed that incubating wild-type *CDC13* cells at 32°C did not lead to a decrease in Cdc5 quantity (Supplementary Fig. 1b). Cdc5 was degraded by the proteasome as evidenced by its stabilization in the presence of the proteasome inhibitor MG132 in the culture media (Supplementary Fig. 1c). The previously identified APC/C-Cdh1-dependent ubiquitinylation motifs, KEN box and destruction box 1 (Charles *et al.* 1998; Arnold *et al.* 2015), as well as Cdh1, the activator of APC/C, did not appear to be involved in this degradation (Supplementary Fig. 1, d–f).

We compared the kinetics of Cdc5 degradation with the kinetics of adaptation in the same strain. To achieve a time-resolved detection of adaptation events, we developed a microfluidics-based assay to monitor cells over 24 h in time-lapse microscopy with the acquisition of 1 image every 10 min (Fig. 1, b and c). Exponentially growing *cdc13-1* cells were loaded into a microfluidic plate as a low-density monolayer in microchambers, set in an incubator at 32°C under the microscope. The chambers were fed with a constant flow of rich media (20 µl/h for 0.036-µl chambers). Multiple fields of view representing >150 cells for each condition or strain were monitored in an automated manner. The cells arrested in G2/M within 2–3 h (Fig. 1, b and c). Similar to the standard microcolony assay used to assess adaptation (Toczyski 2006), we used cell rebudding as an indication that an adaptation event occurred. Only the adaptation of an initially arrested cell was counted as 1 event and we did not count the subsequent cell divisions of adapted cells. In the *CDC5 cdc13-1* strain, the cumulative fraction of adaptation events followed a sigmoid shape with a plateau at 77.0 ± 5.9% (mean ± SD) reached at 16–18 h and half of the adaptation events observed at *t_50_* = 9.36 ± 0.30 h (mean ± SD) (Fig. 1c). This measurement was consistent with the adaptation percentage obtained by microcolony assay (mean ± SD = 75.7 ± 10.2%) (Fig. 1d), where arrested cells were plated at low density and microcolonies containing more than 3 cell bodies were counted after 24 h at 32°C (Toczyski 2006) (Fig. 1e).

The amount of Cdc5 protein in response to telomere dysfunction was inversely correlated to the kinetics of adaptation events, which was surprising since Cdc5 activity is essential for adaptation. We wondered whether this observation could be explained by the increasing fraction of adapted cells entering the next G1 and degrading Cdc5. To test this hypothesis, we performed the same experiment in the adaptation-deficient *cdc5-ad* mutant, which stays blocked in G2/M. We confirmed that this permanent arrest was checkpoint-dependent since cells could divide and form microcolonies in the checkpoint-deficient mutants *Δrad9* and *mec1-21* (Sanchez *et al.* 1996; Desany *et al.* 1998) (Supplementary Fig. 1g). Using our microfluidics assay, we found that virtually no *cdc5-ad* cell underwent adaptation (mean ± SD = 0.6 ± 0.6%) (Fig. 1c). However, the amount of Cdc5-ad protein also decreased in response to telomere dysfunction, suggesting that the decrease of Cdc5 protein level was not due to cells entering the next G1, and potentially occurred in G2/M (Fig. 1f). Consistently, we found that in *cdc13-1* cells arrested at 32°C in the presence of nocodazole or concomitantly with a depletion of Cdc20, both of which preventing cycling past G2/M, Cdc5 also decreased in quantity at 3 and 6 h (see further below Fig. 3b for nocodazole and Supplementary Fig. 3a for the depletion of Cdc20).

### Rad53 inhibits *CDC5* transcription through Ndd1 phosphorylation

*CDC5* belongs to the *CLB2* cluster of genes and their expression depends on the transcription factor Fkh2/Mcm1 associated with the coactivator Ndd1 (Spellman *et al.* 1998). After DDC activation, Ndd1 is phosphorylated by Rad53 and no longer binds Fkh2/Mcm1, thereby limiting the expression of some of these genes (Edenberg *et al.* 2014; Yelamanchi *et al.* 2014). While not all genes in the *CLB2* cluster are repressed in response to DNA damage, the amount of *CDC5* mRNAs was found to be decreased (Gasch *et al.* 2001; Jaehnig *et al.* 2013; Edenberg *et al.* 2014). We therefore asked whether the decrease of Cdc5 at the protein level could reflect a repression of *CDC5* transcription through Rad53-dependent phosphorylation of Ndd1.

**Fig. 2.**
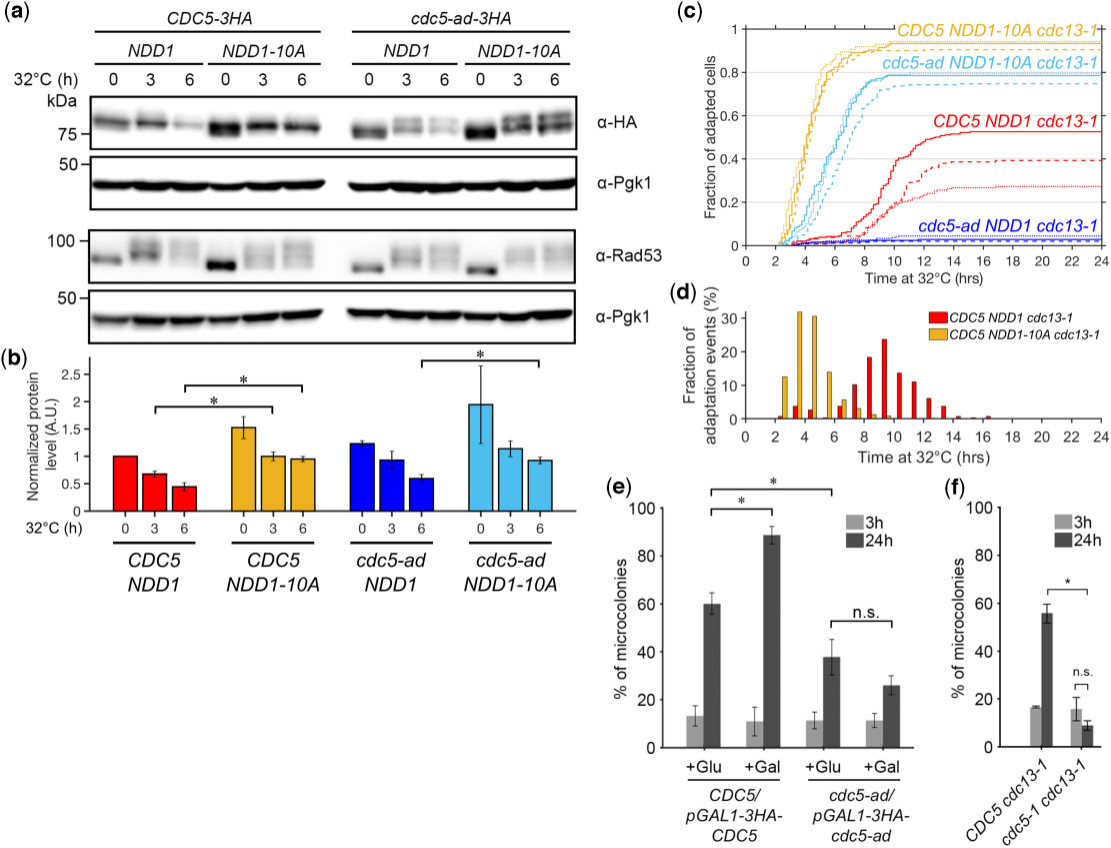
Ndd1 regulates Cdc5 protein level and adaptation kinetics but overexpression of Cdc5-ad does not rescue adaptation defect. a) Representative western blot of Cdc5 and Rad53 in a time course experiment after induction of telomere dysfunction by incubating *cdc13-1* cells at 32°C, in the indicated strains. Pgk1 is shown as a loading control. The presence of the endogenous *NDD1* gene is omitted in the genotype notation for simplicity, for (a)–(d). b) Quantification of the western blot signal for Cdc5 or Cdc5-ad normalized over Pgk1 in 3 independent experiments for the indicated strains incubated at 32°C for 0, 3, and 6 h. Time point 0 for *CDC5 NDD1* is arbitrarily set at 1. Student’s *t*-test statistical significance for *P*-value <0.05 is indicated with “*.” c) Cumulative curves of the fraction of adapted cells as a function of time after induction of telomere dysfunction, in the indicated strains. Three independent experiments for each strain are shown in continuous line, dashed line and dots. d) Distribution of the timing of the adaptation events for the indicated strains, using data shown in (c). e) Microcolony assay measuring the fraction of microcolonies formed in the indicated strains at 3 and 24 h, with or without galactose-induced overexpression of Cdc5/Cdc5-ad. Data are presented as means ± SD of *N* = 3 independent experiments. *n* ≥ 150 cells for each condition. f) Microcolony assay with the thermosensitive allele *cdc5-1* compared to wild type. Data are presented as mean ± SD of *N* = 3 independent experiments. *n* ≥ 150 cells for each condition.

We thus introduced in our strains a second copy of wild-type *NDD1* or mutant *NDD1-CD-10A* allele (called “*NDD1-10A*” hereafter) ectopically expressed from the *LEU2* locus using *NDD1*’s native promoter. The *NDD1-10A* allele is insensitive to DDC-dependent regulation due to 10 mutations removing Rad53 phosphorylation sites (Yelamanchi *et al.* 2014). We chose to keep the endogenous *NDD1* gene to cover for potential pleiotropic phenotypes of the *NDD1-10A* mutant in cell growth for instance. Since Ndd1 is expected to be excluded from promoters in response to telomere dysfunction, keeping the endogenous *NDD1* gene should not prevent the assessment of the dominant effects of Ndd1-10A recruitment. We then monitored *CDC5* or *cdc5-ad* expression at the mRNA level by reverse transcription qPCR and at the protein level by western blot in these strains after inducing telomere deprotection (Fig. 2, a and b and Supplementary Fig. 2a). While CDC5 and *cdc5-ad* transcript levels decreased over time in the *NDD1* strains, they were at least maintained in the *NDD1-10A* strains, a result consistent with a previous study (Edenberg *et al.* 2014). Cdc5 and Cdc5-ad protein levels were higher in *NDD1-10A* strains compared to the *NDD1* strains, but also displayed a gradual decrease (Fig. 2, a and b), suggesting an active degradation mechanism in response to telomere dysfunction. Thus, Rad53-dependent repression of *CDC5* and *cdc5-ad* transcription directly translates into a decrease in protein level, which is partially offset in the *NDD1-10A* strains.

### The Rad53-Ndd1 signaling pathway regulates the kinetics of adaptation

The role of Cdc5 in adaptation is dose dependent and overexpression of Cdc5 accelerates adaptation (Hu *et al.* 2001; Dotiwala *et al.* 2007; Donnianni *et al.* 2010; Vidanes *et al.* 2010), but this property was previously tested with constitutive expression of *CDC5* at different levels (e.g. gene copy number or galactose-inducible promoter). We therefore wondered whether adaptation would also be accelerated in the *NDD1-10A* mutant where Cdc5 was not overexpressed but maintained closer to its initial level. Adding a second copy of wild-type *NDD1* in the *CDC5 cdc13-1* strain affected the final percentage of adapted cells (mean ± SD = 39.8 ± 12.9%), but not the kinetics (*t*_50_ = 9.43 ± 0.57 h, mean ± SD) (Fig. 2c). When *CDC5 cdc13-1* cells expressed *NDD1-10A* as a dominant second copy, adaptation was dramatically accelerated (*t*_50_ = 4.10 ± 0.06 h, mean ± SD) and the total fraction of adapted cells also increased (mean ± SD = 92.3 ± 2.0%) (Fig. 2c). Importantly, we observed that *NDD1-10A* cells arrested in G2/M within the first 2 h, indicating that the checkpoint arrest was functional, consistent with Rad53 being hyperphosphorylated upon induction of telomere deprotection in the *NDD1-10A* mutant (Fig. 2a). Interestingly, at 6 h, ∼90% of *NDD1-10A* cells already adapted but Rad53 was not dephosphorylated, in contrast to the normal expectation after adaptation (Lee *et al.* 2000; Pellicioli *et al.* 2001). This observation suggests that an important step in adaptation driven by Rad53 dephosphorylation might be to allow the Ndd1-dependent activation of the *CLB2* cluster. Alternatively, the *NDD1-10A* mutant possibly allowed adaptation and the bypass of the DDC in a way that might differ from canonical adaptation.

We were also interested in the shape of the adaptation curve in the *NDD1-10A* mutant, which was much steeper than in the *NDD1* control strain when the first cells started to adapt. We thus plotted the distribution of adaptation events over time and confirmed that the distribution in the *NDD1-10A* mutant was narrower and with a positive skew (Fig. 2d). These results are consistent with the idea that the inhibition of the Ndd1-dependent transcription in response to DDC activation, including the downregulation of *CDC5*, strongly contributed to restrain adaptation and to its timing heterogeneity. However, another checkpoint-dependent mechanism prevented cell cycle restart before ∼2 h, as revealed in the *NDD1-10A* mutant.

Strikingly, the *NDD1-10A* allele largely rescued the adaptation defect of *cdc5-ad* whereas a second copy of wild-type *NDD1* did not (fraction of adapted cells at 24 h: mean ± SD = 77.8 ± 2.7% vs 3.1 ± 1.2%, respectively) (Fig. 2c). Adaptation in *cdc5-ad NDD1-10A* cells was slightly slower (*t*_50_ = 5.74 ± 0.39 h, mean ± SD) and appeared slightly less efficient than in *CDC5 NDD1-10A* cells (fraction of adapted cells at 24 h: mean ± SD = 77.8 ± 2.7% vs 92.7 ± 1.9%, respectively) but still accelerated even compared to the *CDC5 NDD1* strain.

We conclude that phosphorylation of Ndd1 by Rad53 in response to telomere dysfunction slows down adaptation kinetics, likely by downregulating Cdc5, and contributes to the adaptation defect of the *cdc5-ad* mutant. Ndd1 phosphorylation is not required for the initial checkpoint arrest but is important for its maintenance after 2 h.

### The transcriptional regulation of the *CLB2* cluster is essential to prevent adaptation in *cdc5-ad*

Since the kinase activity of Cdc5-ad is not impaired (Charles *et al.* 1998; Rawal *et al.* 2016), the relative increase in Cdc5-ad protein level in the *NDD1-10A* mutant might be sufficient to allow adaptation. Alternatively, the checkpoint arrest might be bypassed in the *NDD1-10A* mutant by relieving the global transcriptional inhibition of the *CLB2* cluster of genes, regardless of the *cdc5-ad* allele. To distinguish between these 2 possibilities, we tested whether overexpression of Cdc5-ad alone would be sufficient to rescue *cdc5-ad*’s adaptation defect. Since *CDC5* is an essential gene, we examined adaptation in diploid strains in which expression of only 1 copy of Cdc5 or Cdc5-ad is under the control of a galactose-inducible (and glucose-repressible) promoter and tagged with 3xHA. Upon galactose addition, the 3xHA-tagged Cdc5 and Cdc5-ad were clearly overexpressed within an hour at both permissive and restrictive temperatures (Supplementary Fig. 2b). We then performed microcolony assays to assess the adaptation ability of these strains, by spreading the cells on galactose-containing plates to induce Cdc5 or Cdc5-ad overexpression. We observed that overexpressing Cdc5 increased adaptation efficiency (Fig. 2e), likely by accelerating its kinetics as previously described (Vidanes *et al.* 2010). However, overexpressing Cdc5-ad was not sufficient to recover adaptation capacity (Fig. 2e). To confirm this result in a haploid setting, we expressed *CDC5* or *cdc5-ad* from a 2-µ plasmid and found that while the expression of *CDC5* rescued *cdc5-ad’*s adaptation defect as expected, the expression of *cdc5-ad* did not (Supplementary Fig. 2c). Thus, the adaptation defect of *cdc5-ad* was not simply due to a decreased expression and the Ndd1-dependent regulation of the *CLB2* cluster globally was important to prevent adaptation in the *cdc5-ad* mutant. In contrast to the overexpression of *CDC5*, the temperature-sensitive mutant *cdc5-1*, which shows decreased protein level and activity at restrictive temperature (Cheng *et al.* 1998; Park *et al.* 2003) resulting in a strong growth defect even at 32°C (Chen and Weinreich 2010), failed to adapt to telomere dysfunction (Fig. 2f), reinforcing the notion that adaptation is dose dependent and kinase activity dependent.

### Cdc5-ad is hyperphosphorylated in an Mec1- and Tel1-independent manner

**Fig. 3.**
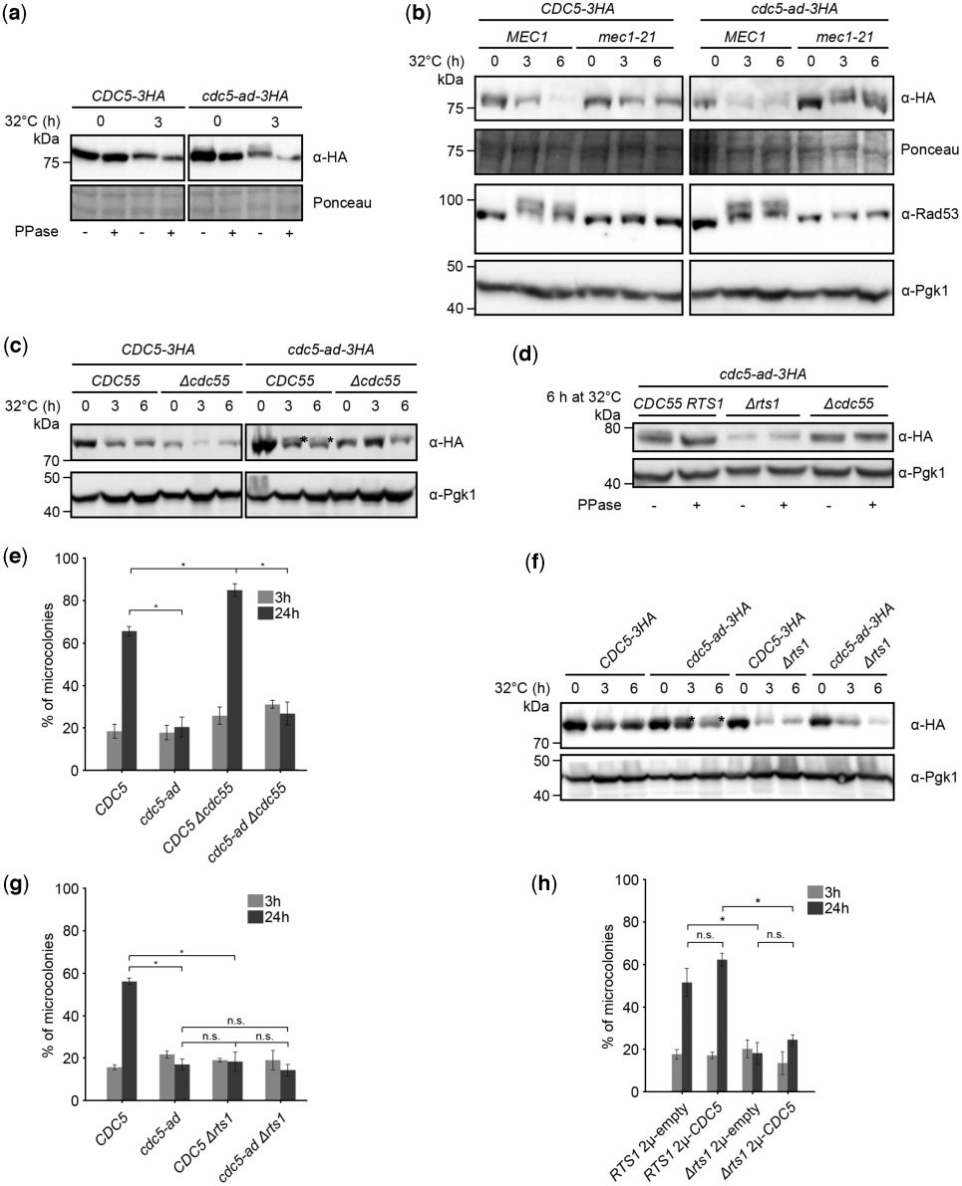
Cdc5-ad is phosphorylated in response to telomere dysfunction in a Mec1- and Tel1-independent and PP2A-dependent manner (see also Supplementary Fig. 3a). a) Representative western blot of Cdc5 or Cdc5-ad from *cdc13-1* cells incubated at 32°C for the indicated amount of time. Samples were treated (+) or not (−) with λ-phosphatase (PPase). b) Representative western blot of Cdc5 or Cdc5-ad and Rad53 in response to telomere dysfunction in a wild-type *MEC1* or *mec1-21* mutant strain, incubated at 32°C for the indicated amounts of time, in the presence of nocodazole. c) Representative western blot of Cdc5 or Cdc5-ad in a wild-type *CDC55* or *Δcdc55* mutant strain. An asterisk indicates the hyperphosphorylated band of Cdc5-ad. d) λ-phosphatase (PPase) assay with samples from the indicated strains incubated at 32°C for 6 h. e) Microcolony assay measuring the fraction of microcolonies formed at 3 and 24 h in the indicated strains. Data are presented as mean ± SD of *N* = 3 independent experiments. *n* ≥ 150 cells for each condition. f) Representative western blot of Cdc5 or Cdc5-ad in a wild-type *RTS1* or *Δrts1* mutant strain. An asterisk indicates the hyperphosphorylated band of Cdc5-ad. g) Microcolony assay measuring the fraction of microcolonies formed at 3 and 24 h in the indicated strains. Data are presented as means ± SD of *N* = 3 independent experiments. *n* ≥ 150 cells for each condition. h) Microcolony assay measuring the fraction of microcolonies formed in the indicated strains at 3 and 24 h, with either an empty 2 µ plasmid (pRS42H) or one expressing *CDC5* under its endogenous promoter. Data are presented as means ± SD of *N* = 3 independent experiments. *n* ≥ 150 cells for each condition. These experiments were performed together with the ones presented in Supplementary Fig. 2c and the 2 control conditions, noted here *RTS1* 2 µ-empty and *RTS1* 2 µ-*CDC5*, are therefore shared between the 2 graphs.

In addition to the transcriptional repression and degradation of Cdc5 and Cdc5-ad in response to telomere dysfunction, we observed that Cdc5-ad migrated as 2 forms in western blot (Fig. 1f). To test whether the slow migrating band corresponds to a phosphorylated form of Cdc5-ad, we treated the samples with λ-phosphatase before electrophoresis and found that the slow migrating band appearing at 3 h was no longer present (Fig. 3a). Interestingly, both Cdc5 and the fast-migrating band of Cdc5-ad also displayed a slight shift in migration when treated with λ-phosphatase, suggesting that these forms were also phosphorylated. Since Cdc5 and Cdc5-ad seemed to be more phosphorylated after telomere dysfunction, we tested whether their phosphorylation depended on the DDC and particularly on Mec1 and Tel1. To do so, we induced telomere dysfunction in the hypomorph mutant *mec1-21* in the presence of nocodazole to prevent cycling beyond G2/M due to checkpoint deficiency. We found by western blot that in the *mec1-21* mutant, Rad53 was no longer hyperphosphorylated, as expected in this checkpoint-deficient mutant, but Cdc5-ad still migrated as 2 bands (Fig. 3b). Because interference with microtubule polymerization by nocodazole can induce spindle assembly checkpoint, we also verified the presence of the double band of Cdc5-ad in *mec1-21* mutant using *CDC20* under the control of a galactose-inducible promoter as a strategy to maintain cells arrested in G2 by depletion of Cdc20 in glucose (Supplementary Fig. 3a). Deletion of *TEL1* also did not affect the double-band migration profile of Cdc5-ad after telomere dysfunction (Supplementary Fig. 3b). Thus, Cdc5-ad-specific phosphorylation did not depend on the Mec1 or the Tel1 branches of the DDC, when mutated separately. However, these 2 mutations had different effects on Cdc5 or Cdc5-ad levels upon telomere dysfunction. Consistent with our results regarding the transcriptional regulation of *CDC5* and *cdc5-ad* by Rad53 and Ndd1, *mec1-21* cells behaved like the *NDD1-10A* mutant and displayed higher levels of Cdc5 and Cdc5-ad (Fig. 3b), while *TEL1* deletion mutant did not (Supplementary Fig. 3b), presumably because the contribution of Tel1 to the DDC signaling is minor compared to Mec1.

### PP2A phosphatases affect adaptation in multiple ways

Since Cdc5 and Cdc5-ad were phosphorylated, particularly in response to telomere dysfunction and more so for Cdc5-ad, we wondered whether phosphatases regulate adaptation. In a recently published time-resolved phosphoproteomic analysis of mitosis (Touati *et al.* 2019), residues T70, T238 and T242 in Cdc5 were found to be more phosphorylated in a PP2A^Rts1^ phosphatase mutant (*Δrts1*) in G2. But dephosphorylation of these residues after mitosis was not delayed, indicating that other phosphatases acted on them. In a PP2A^Cdc55^ phosphatase mutant (*Δcdc55*), dephosphorylation of residue S2 of Cdc5 was delayed in mitosis. To investigate the potential role of *RTS1* and *CDC55* in adaptation, we deleted these genes in our strains and assessed their adaptation phenotype.

Deletion of *CDC55* decreased the overall amount of Cdc5 and Cdc5-ad (Fig. 3c). Surprisingly, the hyperphosphorylated form of Cdc5-ad was no longer present in *Δcdc55* as confirmed by a λ-phosphatase assay (Fig. 3d), suggesting an indirect effect of PP2A^Cdc55^ on Cdc5-ad phosphorylation. In a microcolony assay, we found that the absence of *CDC55* slightly improved the adaptation level in *CDC5 cdc13-1* cells incubated at restrictive temperature (Fig. 3e). This result suggested that Cdc55 counteracted adaptation and that adaptation might normally involve the inhibition or the bypass of Cdc55’s function(s). In the *cdc5-ad cdc13-1* strain, however, deletion of *CDC55* did not rescue the adaptation defect, indicating that the adaptation function affected in *cdc5-ad* is independent of Cdc55.

We then assessed the effect of *RTS1* deletion (Fig. 3, f and g). Strikingly, *RTS1* deletion inhibited adaptation as strongly as the *cdc5-ad* mutant (Fig. 3g). The double mutant *Δrts1 cdc5-ad* was undistinguishable from either *Δrts1* and *cdc5-ad* with respect to adaptation. In the microcolony experiments, *Δrts1 cdc13-1* cells robustly arrested in G2/M after 3 hrs at 32 °C, suggesting that the DDC was fully functional. Furthermore, *Δrts1* cells were proficient in recovery as they grew as well as *RTS1* cells in an assay where cells were subjected to a transient telomere dysfunction (for 10 h) before returning to a permissive temperature (Supplementary Fig. 3c). The adaptation defect of *Δrts1* was not specific to telomere dysfunction, since we found by microcolony assay that in response to a single persistent DSB induced by the I-SceI endonuclease, *Δrts1* cells were also adaptation deficient (Supplementary Fig. 3d). Therefore, *Δrts1* is a new bona fide adaptation mutant. We examined Cdc5 and Cdc5-ad proteins in the *Δrts1* mutant by western blot and found that both proteins started with a similar level but decreased in quantity faster than in a wild-type *RTS1* background (Fig. 3f). To test whether *Δrts1*’s adaptation deficiency was due to an exacerbated decrease of Cdc5 quantity, we expressed *CDC5* in a 2-µ plasmid in the *Δrts1* strain. While the 2-µ-borne *CDC5* was sufficient to rescue *cdc5-ad*’s adaptation deficiency (Supplementary Fig. 2c), it did not increase *Δrts1*’s adaptation level (Fig. 3h), suggesting that *RTS1* might be required for adaptation in a *CDC5*-independent manner. Interestingly, in the *Δrts1* mutant, Cdc5-ad did not show a hyperphosphorylated band (Fig. 3, d and f), indicating that Rts1 is important for Cdc5-ad’s phosphorylation. As for PP2A^Cdc55^, since PP2A^Rts1^ is a phosphatase, the effect on Cdc5-ad’s phosphorylation must be indirect. We conclude that both phosphatases are involved in adaptation and that the hyperphosphorylation of Cdc5-ad is not essential to prevent adaptation.

### Phosphorylation of specific residues in Cdc5 modulates adaptation

**Fig. 4.**
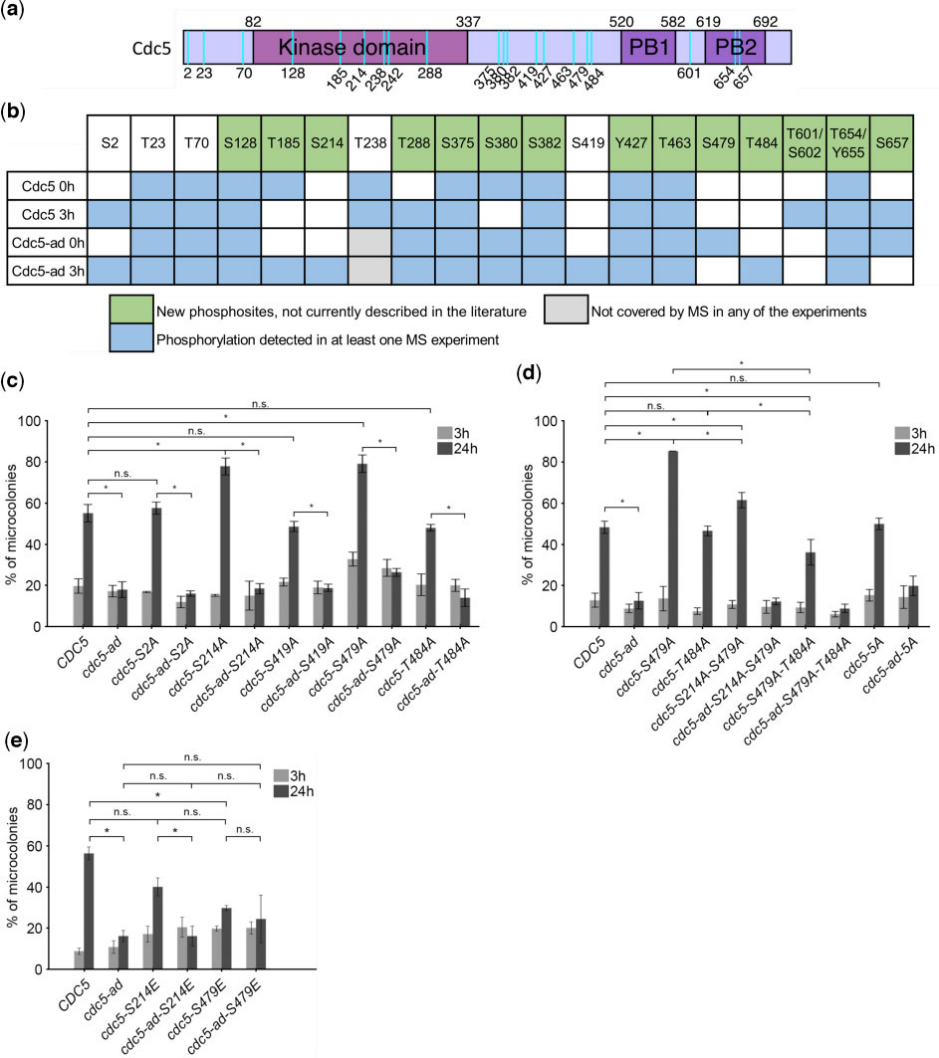
Phosphorylation of Cdc5 modulates adaptation. a) Schematic representation of Cdc5 and its domains (kinase and Polo-box domains PB1 and PB2) with the detected phosphorylation sites mapped (cyan vertical lines). b) Summary table of the putative phosphorylation sites identified by mass spectrometry for each condition. For T601/S602 and T654/Y655, peptide analysis did not allow discrimination between the 2 putative phosphorylation sites. c) Microcolony assay measuring the fraction of microcolonies formed at 3 and 24 h in strains carrying mutations to alanines at the residues S2, S214, S419, S479, and T484. Data are presented as means ± SD of *N* ≥ 3 independent experiments. *n* ≥ 150 cells for each condition. d) Microcolony assay measuring the fraction of microcolonies formed at 3 and 24 h in strains carrying the indicated mutations, including double mutants S214A-S479A, S479A-T484A, and all 5 sites mutated into alanines (“5A”). Data are presented as means ± SD of *N* ≥ 3 independent experiments. *n* ≥ 150 cells for each condition. e) Microcolony assay measuring the fraction of microcolonies formed at 3 and 24 h in *CDC5* and *cdc5-ad* strains carrying the phosphomimetic mutations S214E and S479E. Data are presented as mean ± SD of *N* ≥ 3 independent experiments. *n* ≥ 150 cells for each condition.

To identify the phosphorylated residues in Cdc5 and Cdc5-ad, we immunoprecipitated Cdc5-3HA and Cdc5-ad-3HA in a *cdc13-1* mutant incubated at restrictive temperature for 0 or 3 h, using an anti-HA antibody, and performed mass spectrometry. Although hyperphosphorylation of Cdc5-ad was not essential for adaptation defect, the identification and study of the phosphorylated residues in Cdc5-ad might still be informative. Since the 3-hr samples had less Cdc5 and Cdc5-ad proteins (Fig. 1, a and f), we took advantage of the *NDD1-10A* mutant, in which background both proteins were more abundant at this time point, to perform the experiment. The immunoprecipitated samples were separated by SDS polyacrylamide gel electrophoresis and silver stained (Supplementary Fig. 4, a and b). The bands corresponding to Cdc5 and Cdc5-ad were cut from the gel. Proteins were extracted, trypsinized and analyzed by liquid chromatography coupled to tandem mass spectrometry (LC–MS/MS). Figure 4, a and b summarizes the phosphosites that we detected in 2 independent experiments.

We found that Cdc5 and Cdc5-ad were heavily phosphorylated in vivo, in particular in DDC-arrested cells. Among the 19 phosphosites we detected in at least 1 experiment, 14 have not been described before in the literature, to our knowledge. The phosphosites mapped to all defined domains of Cdc5, including the N-terminus, the kinase domain and the Polo-box domain (PBD) (Fig. 4a).

We were particularly interested in residues that were differentially phosphorylated between Cdc5 and Cdc5-ad or between time points 0 and 3 h. We therefore focused on residues S2, S214, S419 (a minimal Cdk1 site), S479, and T484. We mutated each of these residues to alanine by Cas9-mediated mutagenesis and assessed the adaptation phenotype of the resulting mutant strains. The *cdc5-S214A* and *cdc5-S479A* mutants showed significantly increased adaptation level compared to wild-type without affecting protein level (Supplementary Fig. 4d), suggesting that phosphorylation of these sites inhibited adaptation (Fig. 4c). Other phosphosite mutants did not significantly alter adaptation level (Fig. 4c). Analysis of the double mutant *cdc5-S214A-S479A* showed that, while it still adapted better than the wild-type *CDC5*, it did not further increase adaptation and showed rather less efficient adaptation compared to each individual mutant (Fig. 4d and Supplementary Fig. 4d). Because of the proximity of residues S479 and T484, we tested the effect of their combined mutation. Interestingly, the double mutant S479A T484A had an adaptation level comparable to the single T484A mutant, which was significantly lower than the high adaptation efficiency of S479A. Phosphorylation at S479 and T484 therefore appeared to have opposite effects. While all 5 residues were phosphorylated in *cdc5-ad*, their combined mutation into alanines was not sufficient to alter the double-band migration profile in western blot (Supplementary Fig. 4c) and did not restore adaptation in the *cdc5-ad* mutant (Fig. 4d), although it was not necessarily expected since the hyperphosphorylated form of Cdc5-ad is not essential for its adaptation defect. Since S214A and S479A were the 2 single mutations that affected adaptation level in the *CDC5* strain, we asked whether phosphomimetic mutations of these sites would have the opposite effect. Indeed, the *cdc5-S479E* strain showed a significantly decreased adaptation level, as low as *cdc5-ad-S479E*, without affecting protein level (Supplementary Fig. 4d), indicating that it is an adaptation mutant of *CDC5* (Fig. 4e). The adaptation level of *cdc5-S214E* was not significantly different from *cdc5-S479E*, suggesting that it might also be slightly impaired for adaptation (Fig. 4e and Supplementary Fig. 4d). However, the difference between *cdc5-S214E* and wild-type *CDC5* was not significant, although the *P*-value from the 2-sided *t*-test was only 0.059. Despite several attempts, we were not able to generate the *cdc5-T484E* mutant by CRISPR/Cas9-mediated editing, which might perhaps be explained by a potential lethality conferred by this phosphomimetic mutant.

Overall, our results suggested that phosphorylation of single residues such as S214 and S479 inhibits adaptation. The combination with the phosphorylation status of other residues revealed a complex phosphorylation pattern modulating adaptation efficiency or timing. However, the adaptation deficiency of *cdc5-ad* did not solely depend on these phosphorylated residues.

### Cdc5’s functions in different pathways are important for adaptation

**Fig. 5.**
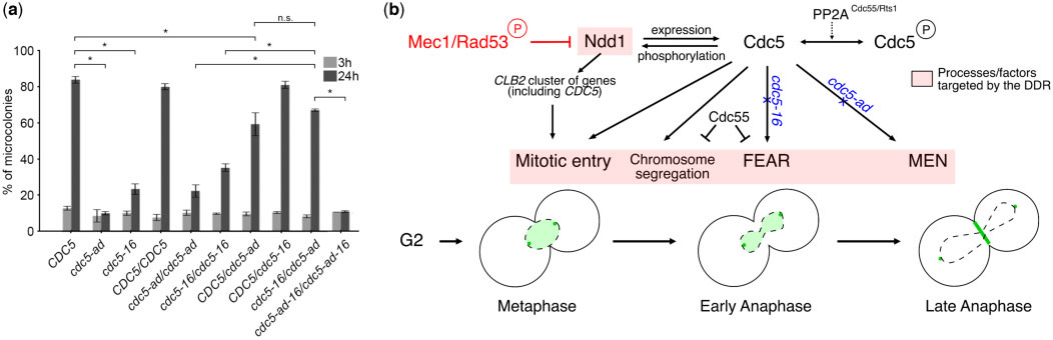
a) Mutant alleles *cdc5-16* and *cdc5-ad* complement each other in adaptation. Microcolony assay measuring the fraction of microcolonies formed at 3 and 24 h in the indicated mutants. Data are presented as means ± SD of *N* = 3 independent experiments. *n* ≥ 150 cells for each condition. b) Model recapitulating the network of regulation of Cdc5 involved in adaptation to DNA damage. The DDR and its Mec1-Rad53 branch target a number of late cell cycle processes and factors (pink shaded boxes), including Ndd1, to ensure robust arrest. In response to telomere dysfunction, Cdc5 is regulated at multiple levels, by Ndd1, by degradation and by phosphorylation/dephosphorylation. The role of PP2A^Cdc55/Rts1^ phosphatases in regulating Cdc5 phosphorylation is depicted with a dashed line as it is indirect. In turn, Cdc5 targets many of the processes “locked” by the DDR (pink shaded boxes), as exemplified by mutant alleles *cdc5-16* and *cdc5-ad* deficient in specific pathways (alleles in blue with blue crosses to indicate deficiency), thus coordinating cell cycle restart. Cdc5 localization in the nucleus, at the spindle pole bodies and at the budneck, in metaphase and anaphase, is shown in green.

Since Cdc5 regulates multiple late cell cycle events, any of these could potentially be involved in Cdc5’s role in adaptation. For instance, the deletion of *BFA1*, the gene encoding the mitotic exit substrate of Cdc5, rescues the adaptation defect of *cdc5-ad* (Rawal *et al.* 2016). Cdc5’s interaction with other substrates, such as the RSC complex implicated in the “Cdc-Fourteen Early Anaphase Release” (FEAR) pathway (Rossio *et al.* 2010), is also important for adaptation, as evidenced by the adaptation defect of the *cdc5-16* mutant, which is mutated at 3 amino acids in the PBD, preventing its canonical interaction with primed substrates, including the RSC complex (Ratsima *et al.* 2016). We therefore asked whether these 2 *CDC5* mutants, *cdc5-ad* and *cdc5-16*, had the same underlying molecular defect in adaptation or whether they are affected in distinct pathways. We tested their genetic interaction by introducing these two alleles in diploid *cdc13-1/cdc13-1* cells and assessed the adaptation efficiency of this strain by microcolony assay (Fig. 5a). As a control, we also combined the mutations of *cdc5-16* with the point mutation (L251W) of *cdc5-ad* to generate the *cdc5-ad-16* mutant.

A wild-type copy of *CDC5* was sufficient to rescue the adaptation defect of *cdc5-ad* and *cdc5-16*. Strikingly, *cdc5-ad* and *cdc5-16* were able to complement each other’s adaptation deficiency as the heterozygous *cdc5-ad/cdc5-16* was adaptation proficient (Fig. 5a). In contrast, the *cdc5-ad-16/cdc5-ad-16* mutant was as adaptation deficient as *cdc5-ad/cdc5-ad*. We conclude that *cdc5-ad* and *cdc5-16* are affected in adaptation in different pathways or at least at different steps of the same pathway.

## Discussion

### Regulation of Cdc5 protein level in response to telomere dysfunction

Cdc5 was identified among the downstream targets of the DDC and proposed to be inhibited to prevent anaphase entry and mitotic exit in response to DNA damage (Sanchez *et al.* 1999). While a Rad53- and Ndd1-dependent inhibition of *CDC5* transcription was reported previously (Gasch *et al.* 2001; Jaehnig *et al.* 2013; Edenberg *et al.* 2014), how Cdc5 protein levels are regulated in response to telomere dysfunction has not been clearly established, especially as a function of time. Indeed, the temporal dimension of Cdc5 regulation is particularly important with respect to adaptation, which occurs late after the initial damage (4–16 h) but depends on Cdc5 activity.

Here, we find that Cdc5 protein level decreases progressively in G2/M-arrested cells after telomere dysfunction in a Mec1-, Rad53-, and Ndd1-dependent manner, as a result of the transcriptional repression of *CDC5* and proteasome-dependent degradation (Fig. 5b). Incidentally, after telomere dysfunction, the previously described APC/C-Cdh1-dependent ubiquitinylation of Cdc5 KEN box and destruction box 1 (Charles *et al.* 1998; Arnold *et al.* 2015) is not involved. In a normal cell cycle, APC/C-Cdh1 starts to be active at the end of anaphase and in telophase and would not mediate Cdc5 degradation at the metaphase to anaphase transition. However, it was reported that Cdh1 is maintained in an active state in G2/M by the DDC in *cdc13-1* cells at restrictive temperature to prevent chromosome segregation (Zhang *et al.* 2009). The question of the mechanisms underlying Cdc5 degradation in this context thus remains to be investigated.

Cdc5 being essential for adaptation, it might be surprising to observe that Cdc5 degradation after telomere dysfunction follows a kinetics that is inversely correlated to that of adaptation. One possibility would be that late cell cycle events are normally executed with an excess of Cdc5 proteins, consistently with the fact that a kinase mutant (*cdc5-77*) with less than 2% of wild-type activity is still viable (Ratsima *et al.* 2011). This excess ensures a robust cell cycle progression but might be incompatible with checkpoint arrest. The decrease in Cdc5 quantity after telomere dysfunction would then be an essential step to enforce cell cycle control by the DDC. The low amount of Cdc5 might still be enough to trigger adaptation though, even after a long delay of 8–12 h. Cdc5’s activity in these conditions would then be finely controlled by other mechanisms such as posttranslational modifications, as our phosphosite mutagenesis results suggest. In this model, alterations in Cdc5 levels would tip the balance of Cdc5’s fine-tuned activity, thus explaining the many results correlating adaptation efficiency with the total amount of Cdc5, as we see in *NDD1-10A*, *Δrts1*, and strains overexpressing Cdc5, or as described previously (Hu *et al.* 2001; Dotiwala *et al.* 2007; Donnianni *et al.* 2010; Vidanes *et al.* 2010). An exception to this rule would be the *Δcdc55* mutant in which adaptation is promoted while Cdc5 level is lower than in wild-type cells. In this case, the role of Cdc55 in preventing sister chromatid separation and inhibiting mitotic exit in the presence of DNA damage (Tang and Wang 2006; Liang and Wang 2007) might explain the slightly more efficient adaptation of the *Δcdc55* mutant.

Alternatively, adaptation might be triggered by a transient burst of Cdc5 activity or quantity at the single cell level. Such a transient increase in activity or quantity would not be detected by measuring the total amount of Cdc5 at the population level, given the variability of adaptation timing at the single cell level. The transient and heterogeneous behavior of Cdc5 expression would be consistent with the positive feedback regulation of Cdc5 on Ndd1 (Fig. 5b), whereby Cdc5 phosphorylates Ndd1 on residue S85 to promote its binding to the promoter of the *CLB2* cluster of genes, thus stimulating its own expression (Darieva *et al.* 2006). Since Rad53 phosphorylates both Ndd1 and Cdc5, and Cdc5 can in turn phosphorylate Rad53 and Ndd1 (Darieva *et al.* 2006; Zhang *et al.* 2009; Vidanes *et al.* 2010; Edenberg *et al.* 2014; Yelamanchi *et al.* 2014), the dynamic output of this genetic network is complex but might have been harnessed to control adaptation timing and heterogeneity.

A third possibility would be that the DDR simultaneously promotes Cdc5’s activity toward substrates relevant for adaptation and triggers its downregulation and degradation. In addition, Cdc5 might function early after the initial damage when its level is still high but its actual effect on adaptation might take several hours, potentially due to signal transduction through one or several pathways and to mechanisms that temporarily block adaptation progression (see below). There would then be a large delay between Cdc5’s peak activity after DNA damage and adaptation timing. This scenario would also be compatible with the observations correlating Cdc5 dosage with adaptation rate and efficiency.

### Posttranslational modifications of Cdc5

Phosphorylation of Cdc5 and of Polo kinases in general is important for their kinase activity (Hamanaka *et al.* 1995; Tavares *et al.* 1996; Cheng *et al.* 1998; Qian *et al.* 1998; Mortensen *et al.* 2005; Rawal *et al.* 2016; Rodriguez-Rodriguez *et al.* 2016; Li *et al.* 2019). In addition, Cdc5 is specifically phosphorylated in response to DNA damage in a Mec1- and Rad53-dependent manner (Cheng *et al.* 1998; Zhang *et al.* 2009). We also find that Cdc5 is phosphorylated after telomere dysfunction but most strikingly, we report that in this context Cdc5-ad migrates as 2 bands with distinct electrophoretic mobility. We note that, although it is induced by telomere deprotection, the slow migrating form of Cdc5-ad does not depend on Mec1 and Tel1 and is not directly due to the cells being in G2/M since a previous study showed that Cdc5-ad does not display an electrophoretic mobility shift during the cell cycle (Rawal *et al.* 2016).

Adaptation assays performed in mutants of individual sites (S214 and S479) indicate that their phosphorylation limits adaptation. We thus suggest that in addition to the downregulation of protein level, phosphorylation of Cdc5 might constitute another layer of regulation to prevent premature adaptation (Fig. 5b). However, combinatorial phosphorylation pattern of Cdc5 can be more complex since we evidence opposing effects of different phosphorylated sites, using combined mutants of 2 or more residues. None of the mutations we tested or their combinations rescued, even partially, the adaptation defect of *cdc5-ad*. Even the mutation of 5 selected residues did not affect the adaptation phenotype of *cdc5-ad* nor the electrophoretic migration pattern of Cdc5-ad. The function and regulation of Cdc5-ad’s hyperphosphorylation, although not essential for adaptation defect, remain to be investigated. We speculate that other phosphorylations of Cdc5 that were not covered by the peptides detected by MS might also participate in adaptation regulation.

Since the dynamics of dephosphorylation of Cdc5 residues were recently shown to be slightly altered in PP2A phosphatases mutants (Touati *et al.* 2019), we asked whether these phosphatases played a role in the electrophoretic mobility of Cdc5 and Cdc5-ad and in adaptation. Remarkably, both *Δcdc55* and *Δrts1* affect Cdc5/Cdc5-ad abundance and phosphorylation, and adaptation, but in very different ways. *Δrts1* cells display a strong adaptation defect unrelated to Cdc5 protein level, without affecting recovery after transient telomere dysfunction. Importantly, the adaptation defect of *Δrts1* mutant was observed in response to both telomere dysfunction and a single DSB, indicating that the *Δrts1* mutant has a general adaptation deficiency rather than a damage-specific phenotype. In contrast, *Δcdc55* cells adapt slightly more efficiently, probably because cells must bypass the functions of Cdc55 in inhibiting Cdc14 nucleolar release in the FEAR pathway and in preventing chromosome segregation (Queralt *et al.* 2006; Clift *et al.* 2009; Yaakov *et al.* 2012) (Fig. 5b). Since *Δcdc55 cdc5-ad* cells do not adapt, we infer that *cdc5-ad* is not defective in FEAR and chromosome segregation after DNA damage or at least not only in these 2 pathways. Strikingly, the slow migrating form of Cdc5-ad is not present in the absence of *CDC55* or *RTS1*, suggesting an indirect regulation of Cdc5-ad phosphorylation by PP2A phosphatases, again showing the complexity of the posttranslational regulation of Cdc5.

### Multiple Cdc5-dependent pathways control adaptation

Cdc5 is involved in many cell cycle related processes (Botchkarev and Haber 2018). Their coordination is important not only for a normal cell cycle but also during adaptation to ensure correct cell division despite persistent damage. Which of them is the limiting one or the first to be unblocked in adaptation and whether multiple Cdc5-dependent processes need to be overcome for adaptation remain unclear.

In the first studies characterizing the DDC status during adaptation, Rad53 phosphorylation and kinase activity were shown to mirror cell cycle arrest, and adaptation correlated well with the return of Rad53 to an unphosphorylated state (Lee *et al.* 2000; Pellicioli *et al.* 2001). Consistently, in adaptation mutants including *cdc5-ad*, Rad53 activation persisted for as long as 24 h (Pellicioli *et al.* 2001). Since then, 2 adaptation defective mutants of *CDC5*, *cdc5-16* and *cdc5-T238A*, have illustrated an uncoupling between Rad53 status and adaptation (Ratsima *et al.* 2016; Rawal *et al.* 2016). In both cases (particularly obvious in *cdc5-16* cells), Rad53 returns to an unphosphorylated state while the cells stay arrested for much longer. Here, on the other hand, we find that in the *NDD1-10A* mutant, most cells have adapted within 4–6 h of telomere deprotection while Rad53 is still hyperphosphorylated. Rad53 dephosphorylation is therefore neither necessary nor sufficient for adaptation, although in a wild-type context, it is likely the most straightforward way for the cell to alleviate the checkpoint arrest during adaptation. A direct phosphorylation of Rad53 by Cdc5 might contribute to checkpoint deactivation (Schleker *et al.* 2010; Vidanes *et al.* 2010). While we suggest that Rad53 phosphorylation can be uncoupled from adaptation, the DDR does target a number of cell cycle progression steps to ensure a robust arrest (Fig. 5b). We propose that they constitute “locking mechanisms” that need to be bypassed for adaptation to occur. Based on our results and on insights from literature, we develop below the idea that Cdc5 appears to target many of them to orchestrate adaptation (Fig. 5b).

The initial checkpoint arrest does not require a functional Ndd1-dependent regulation of the *CLB2* cluster, as it is still present in the *NDD1-10A* mutant, and represents a first locking mechanism. The effect of the *NDD1-10A* mutant also demonstrates that, downstream of Rad53 activation, the transcriptional regulation of the *CLB2* cluster of genes is important for the long-term maintenance of the checkpoint arrest after 2–3 h and therefore represents another locking mechanism for adaptation.

We also suggest that Cdc55 restrains adaptation through its functions in the FEAR network and in preventing chromosome segregation (Fig. 5b) (Queralt *et al.* 2006; Yaakov *et al.* 2012), both of which antagonize Cdc5’s role in these processes. As noted before, the adaptation defect of *cdc5-ad* is independent of Cdc55 and must be caused by yet another locking mechanism, possibly the failure to activate MEN (Fig. 5b) (Rawal *et al.* 2016). In addition, we show that 2 adaptation mutants of *CDC5*, *cdc5-ad* and *cdc5-16*, can complement each other, demonstrating that multiple pathways controlled by Cdc5 are blocked simultaneously during checkpoint arrest and that Cdc5 needs to act on them all.

In conclusion, our work shows that multiple levels of regulation of Cdc5 in response to telomere dysfunction are involved in long-term checkpoint arrest, which requires blocking several processes related to cell cycle progression. Conversely, restarting all of them in a coordinated manner during adaptation is a complicated task for the cell. It is therefore remarkable that Cdc5 is implicated in many of these processes, if not all, supporting the idea that Cdc5 orchestrates, during adaptation, the multiple late cell cycle events leading to completion of mitosis.

## Supplementary Material

iyac171_Supplementary_Data

## Data Availability

Strains and plasmids are available upon request. The authors affirm that all data necessary for confirming the conclusions of the article are present within the article, figures, and tables. Supplemental material is available at GENETICS online.
