# Supplementary material for: The Polo kinase Cdc5 is regulated at multiple levels in the adaptation response to telomere dysfunction
Source: Genetics. 2022 Nov 7;223(1):iyac171. doi: 10.1093/genetics/iyac171 (PMC9836022; doi:10.1093/genetics/iyac171)
Supplement: iyac171_Supplementary_Data [file iyac171_supplementary_data.pdf]

Supplementary Data

Title: The Polo kinase Cdc5 is regulated at multiple levels in the adaptation response to telomere dysfunction

Authors: Héloïse Coutelier[1,2,*], Oana Ilioaia[2,*], Jeanne Le Peillet[2,*], Marion Hamon[3], Damien D'Amours[4], Maria Teresa Teixeira[1], Zhou Xu[2].

Strain	Genotype
yT973 [1, 3]	MATa cdc5::CDC5-3HA-TRP1
yT851 [1]	MATa cdc5::CDC5-3HA-TRP1 cdc13-1
yT853 [1]	MATa CDC5 cdc13-1
yT855 [1]	MATa cdc5::cdc5-ad-3HA-TRP1 cdc13-1
yT1404 [1]	MATa cdc5::cdc5-ad cdc13-1
yZX055 [2]	MATa/α CDC5/cdc5::HIS3MX6-pGAL1-3HA-CDC5 cdc13-1/cdc13-1
yZX056 [2]	MATa/α cdc5::cdc5-ad/cdc5::HIS3MX6-pGAL1-3HA-cdc5-ad cdc13-1/cdc13-1
yZX157 [1]	MATa mec1::mec1-21 cdc5::CDC5-3HA-TRP1 cdc13-1
yZX159 [1]	MATa mec1::mec1-21 cdc5::cdc5-ad-3HA-TRP1 cdc13-1
yZX060 [1]	MATa rts1::LEU2 cdc5::CDC5-3HA-TRP1 cdc13-1
yZX063 [1]	MATa rts1::LEU2 cdc5::cdc5-ad-3HA-TRP1 cdc13-1
yZX034 [1]	MATa cdc55::LEU2 cdc5::CDC5-3HA-TRP1 cdc13-1
yZX036 [1]	MATa cdc55::LEU2 cdc5::cdc5-ad-3HA-TRP1 cdc13-1
yZX196 [1]	MATa tel1::LEU2 cdc5::CDC5-3HA-TRP1 cdc13-1
yZX194 [1]	MATa tel1::LEU2 cdc5::cdc5-ad-3HA-TRP1 cdc13-1
yT1320 [1]	MATa leu2::NDD1-3HA-LEU2 cdc5::CDC5-3HA-TRP1 cdc13-1
yT1322 [1]	MATa leu2::ndd1-CD-10A-3HA-LEU2 cdc5::CDC5-3HA-TRP1 cdc13-1
yT1324 [1]	MATa leu2::NDD1-3HA-LEU2 cdc5::cdc5-ad-3HA-TRP1 cdc13-1
yT1326 [1]	MATa leu2::ndd1-CD-10A-3HA-LEU2 cdc5::cdc5-ad-3HA-TRP1 cdc13-1
yT1120 [2, 3]	MATa CDC5 cdc13-1
yT1124 [2,3]	MATa cdc5::cdc5-ad-3HA-TRP1 cdc13-1
yT1122 [2, 3]	MATa cdc5::cdc5-16-HIS3MX6 cdc13-1
yT1115 [2, 3]	MATa/α CDC5/CDC5 cdc13-1/cdc13-1
yT1119 [2, 3]	MATa/α cdc5::cdc5-16-HIS3MX6/cdc5::cdc5-16-HIS3MX6 cdc13-1/cdc13-1
yT1113 [2, 3]	MATa/α CDC5/cdc5::cdc5-16-HIS3MX6 cdc13-1/cdc13-1
yT1130 [2, 3]	MATa/α cdc5::cdc5-ad-3HA-TRP1/cdc5::cdc5-16-HIS3MX6 cdc13-1/cdc13-1
yT1126 [2]	MATa/α cdc5::cdc5-ad-3HA-TRP1/cdc5::cdc5-ad-3HA-TRP1 cdc13-1/cdc13-1
yT1128 [2, 3]	MATa/α CDC5/cdc5::cdc5-ad-3HA-TRP1 cdc13-1/cdc13-1
yT1231 [2, 3]	MATa/α cdc5::cdc5-ad-16-HIS3MX6/cdc5::cdc5-ad-16-HIS3MX6 cdc13-1/cdc13-1
yZX090 [1]	MATa cdc5::cdc5-S214A-3HA-TRP1 cdc13-1
yZX139 [1]	MATa cdc5::cdc5-ad-S214A-3HA-TRP1 cdc13-1
yZX064 [1]	MATa cdc5::cdc5-S2A-3HA-TRP1 cdc13-1
yZX067 [1]	MATa cdc5::cdc5-ad-S2A-3HA-TRP1 cdc13-1
yZX095 [1]	MATa cdc5::cdc5-S419A-3HA-TRP1 cdc13-1
yZX097 [1]	MATa cdc5::cdc5-ad-S419A-3HA-TRP1 cdc13-1
yZX094 [1]	MATa cdc5::cdc5-S479A-3HA-TRP1 cdc13-1
yZX092 [1]	MATa cdc5::cdc5-ad-S479A-3HA-TRP1 cdc13-1
yZX046 [1]	MATa cdc5::cdc5-T484A-3HA-TRP1 cdc13-1
yZX048 [1]	MATa cdc5::cdc5-ad-T484A-3HA-TRP1 cdc13-1
yZX190 [1]	MATa cdc5::cdc5-S214A-S479A-3HA-TRP1 cdc13-1
yZX187 [1]	MATa cdc5::cdc5-ad-S214A-S479A-3HA-TRP1 cdc13-1
yZX192 [1]	MATa cdc5::cdc5-S479A-T484A-3HA-TRP1 cdc13-1
yZX189 [1]	MATa cdc5::cdc5-ad-S479A-T484A-3HA-TRP1 cdc13-1
yZX141 [1]	MATa cdc5::cdc5-S2A-S214A-S419A-S479A-T484A-3HA-TRP1 cdc13-1
yZX142 [1]	MATa cdc5::cdc5-ad-S2A-S214A-S419A-S479A-T484A-3HA-TRP1 cdc13-1
yZX244 [1]	MATa cdc5::cdc5-S214E-3HA-TRP1 cdc13-1
yZX246 [1]	MATa cdc5::cdc5-ad-S214E-3HA-TRP1 cdc13-1
yZX248 [1]	MATa cdc5::cdc5-S479E-3HA-TRP1 cdc13-1
yZX250 [1]	MATa cdc5::cdc5-ad-S479E-3HA-TRP1 cdc13-1
yZX252 [1]	MATa cdc5::cdc5-ad-3HA-TRP1 cdc13-1 KanMX6-pGAL1-3HA-CDC20
yZX258 [1]	MATa mec1::mec1-21 cdc5::cdc5-ad-3HA-TRP1 cdc13-1 KanMX6-pGAL1-3HA-CDC20
yZX114 [1]	MATa cdh1::LEU2 cdc13-1
yZX078 [1]	MATa cdc5::cdc5-K35A-E36A-3HA cdc13-1
yZX081 [1]	MATa cdc5::cdc5-ad-K35A-E36A-3HA cdc13-1
yZX131 [1]	MATa cdc5::cdc5-K35A-E36A-R17A-L20A-3HA cdc13-1
yZX133 [1]	MATa cdc5::cdc5-ad-K35A-E36A-R17A-L20A-3HA cdc13-1
yKD516	MATa lys2::ura3-ISceI(lox) rap1::GFP-RAP1-LEU2 hmLΔ::HPH trp1::pGal-IsceI-TRP1 ura3-1::KanMX
yZX310	yKD516 rts1::HIS3MX6
yZX317 [1]	MATa cdc5::CDC5-3HA-TRP1 cdc13-1 rad9::LEU2
yZX319 [1]	MATa cdc5::cdc5-ad-3HA-TRP1 cdc13-1 rad9::LEU2
yZX323	MATa cdc5-1 cdc13-1

[1]strains that carry *TLC1* under a doxycycline-repressible *pTetO2* promoter which provides for active telomerase in the absence of doxycycline (see Xu et al., 2015). All others have wild-type *TLC1*.

[2]strains that carry the fluorescent reporter construct *ura3-1::caURA3-pRPS20-FHAIdomain-mCherry* used to confirm checkpoint activation in microscopy experiments (see Coutelier et al., 2018).

[3]strains that carry the fluorescent nuclear marker *hta2::HTA2-ECFP-KANMX6* (see Coutelier et al., 2018).

Supplementary Table 1. Yeast strains used in this study.

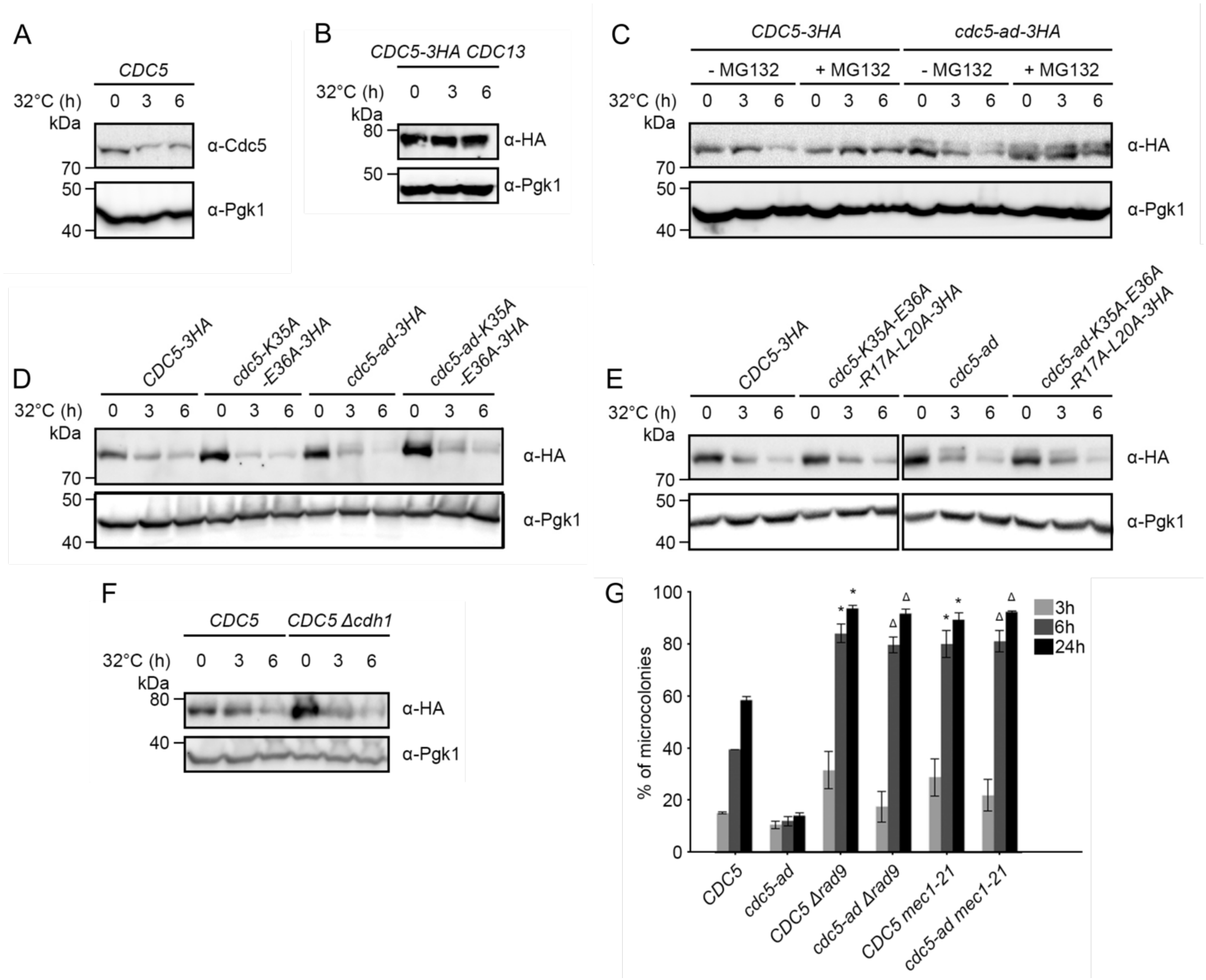

Supplementary Figure S1. (A) Representative western blot of untagged Cdc5 in the *cdc13-1* mutant incubated for the indicated times at 32°C. Pgk1 is shown as a loading control. **(B)** Representative western blot of Cdc5 in the wild-type *CDC13* strain subjected to a temperature of 32°C for the indicated times. **(C)** Representative western blot of Cdc5 and Cdc5-ad in the *cdc13-1* mutant incubated for the indicated times at 32°C, with or without MG132. **(D)** Representative western blot of Cdc5 and Cdc5-ad, with or without additional K35A E36A mutations in the KEN box. The strains all contain the *cdc13-1* allele and were incubated for the indicated times at 32°C. **(E)** Representative western blot of Cdc5 and Cdc5-ad, with or without additional K35A E36A mutations in the KEN box and R17A L20A in the destruction box 1. The strains all contain the *cdc13-1* allele and were incubated for the indicated times at 32°C. **(F)** Representative western blot of Cdc5, in *cdc13-1* or *cdc13-1 cdh1Δ* strains. The strains were incubated for the indicated times at 32°C. **(G)** Microcolony assay measuring the fraction of microcolonies formed in the indicated strains at 3, 6 and 24 hrs. Data are presented as means ± SD of N = 3 independent experiments. n ≥ 150 cells for each condition. Statistical significance for p-value < 0.05 is indicated with "*" when compared to *CDC5* and with "Δ" when compared to *cdc5-ad*, for the corresponding time point.

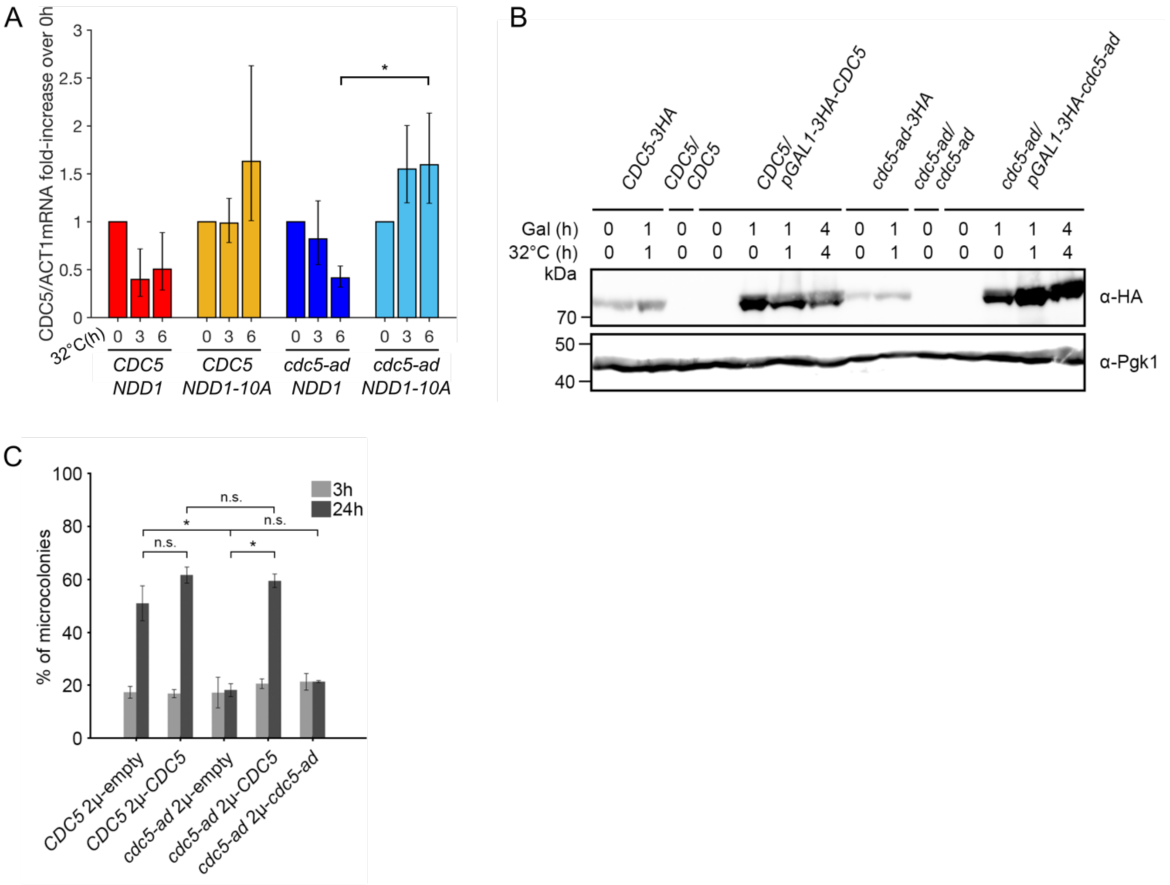

Supplementary Figure S2. (A) Quantification of *CDC5* mRNA level normalized to *ACT1* measured by RT-qPCR and presented as fold increase over time point 0, in the indicated strains incubated at 32°C for 0, 3 and 6 hrs. The ΔΔCt method was used for quantification of 3 independent experiments and the mean and lower/higher ranges for SD, as well as statistical significance from a t-test (*: p-value < 0.05), were calculated at the Ct level before log transformation. **(B)** Representative western blot of Cdc5 with or without overexpression in the indicated *cdc13-1* diploid and haploid strains. Pgk1 is shown as a loading control. **(C)** Microcolony assay measuring the fraction of microcolonies formed in the indicated strains at 3 and 24 hrs, with either an empty 2µ plasmid (pRS42H) or one expressing *CDC5* or *cdc5-ad* under their endogenous promoter, as indicated. Data are presented as means ± SD of N = 3 independent experiments. n ≥ 150 cells for each condition. These experiments were performed together with the ones presented in Fig. 3H and the two control conditions, *CDC5* 2µ-empty and *CDC5* 2µm-*CDC5*, are therefore shared between the two graphs.

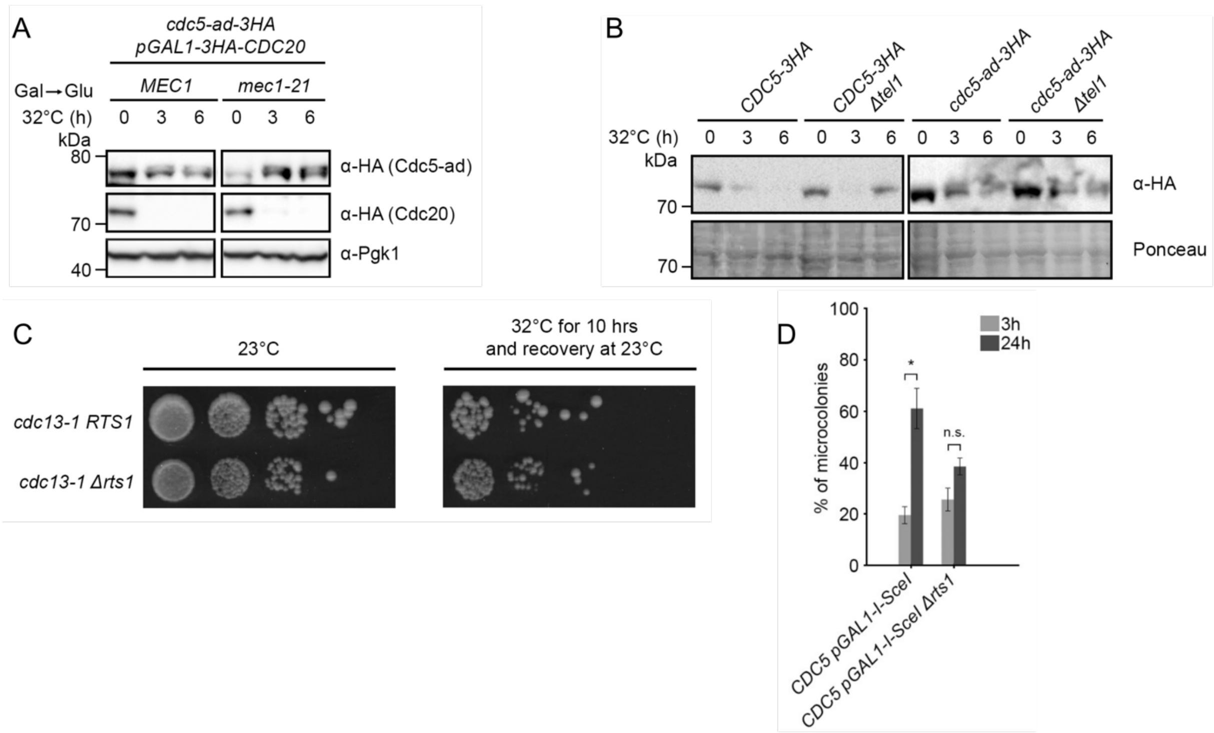

Supplementary Figure S3. (A) Representative western blot of Cdc5-ad in response to telomere dysfunction in a wild-type *MEC1* or *mec1-21* mutant strain, incubated at 32°C for the indicated amounts of time and simultaneously arrested in G2 by depletion of Cdc20. **(B)** Representative western blot of Cdc5 and Cdc5-ad in the *cdc13-1 TEL1* or *Δtel1* strains, incubated for the indicated times at 32°C, in the presence of nocodazole. The increase in Cdc5 protein level at 6 hrs compared to 3hrs in the *Δtel1* strain is reproducible. **(C)** Recovery assay in *cdc13-1 RTS1* or *Δrts1* strains. Ten-fold serial dilutions of cells were incubated at 32°C for 10 hrs to induce transient telomere dysfunction and allowed to recover at 23°C afterwards, or continuously grown at 23°C as a control. **(D)** Microcolony assay measuring the fraction of microcolonies formed in the indicated strains at 3 and 24 hrs, first incubated in liquid galactose medium for 3 hrs before plating on galactose-containing solid media to induce a single DSB by I-SceI expression. Data are presented as means ± SD of N = 3 independent experiments. n ≥ 150 cells for each condition.

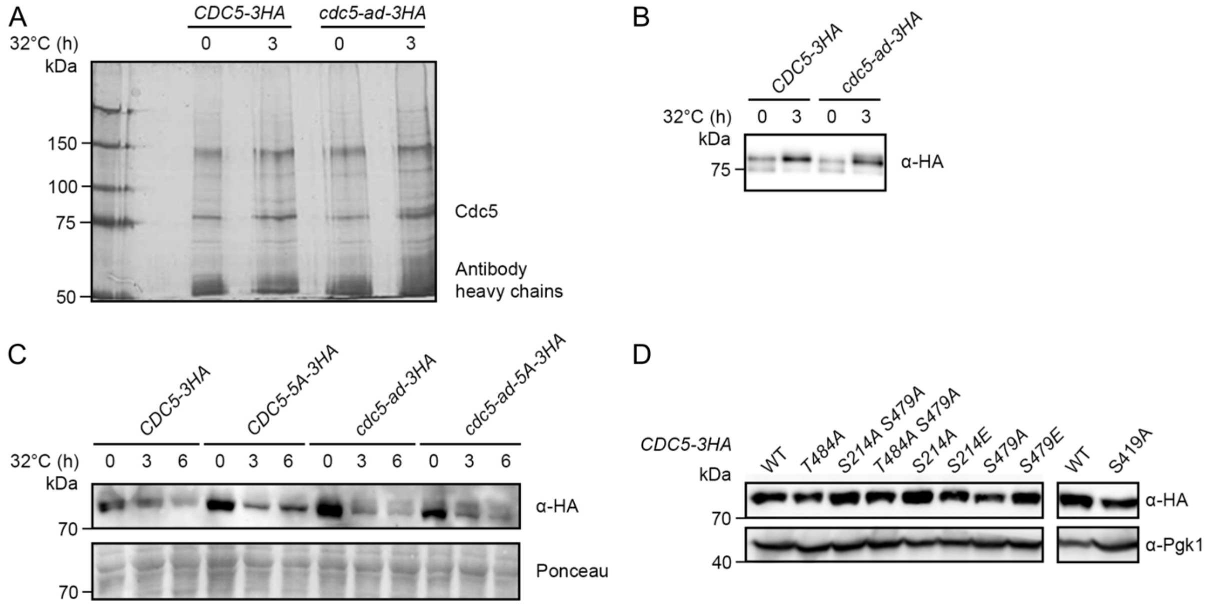

Supplementary Figure S4. (A) Silver-nitrate-stained gel showing immunoprecipitation products from *ndd1-10A cdc13-1* strains incubated for the indicated amounts of time at 32°C, which were then cut out for mass spectroscopy analysis. **(B)** Western blot of the immunoprecipitated Cdc5 and Cdc5-ad from *ndd1-10A cdc13-1* strains incubated at 32°C for the indicated amount of time. **(C)** Representative western blot of Cdc5 and Cdc5-ad, with or without additional mutation of all 5 phosphosites into alanines ("5A"), in the *cdc13-1* strain, incubated for the indicated times at 32°C. **(D)** Representative western blot showing the expression level of wild-type Cdc5 and indicated mutants.
